# The first two cases of posterior reversible encephalopathy syndrome (PRES) secondary to conventional transcatheter arterial chemoembolization of hepatocellular carcinoma

**DOI:** 10.1186/s12876-021-02069-w

**Published:** 2021-12-20

**Authors:** Kefeng Jia, Weili Yin, Fang Wang, Zhongsong Gao, Cheng Sun, Hui Wang, Yujuan Han, Yongmei Wang, Mingge Li, Changlu Yu

**Affiliations:** 1 Department of Radiology, Tianjin Third Central Hospital, No. 83, Jintang Road, Hedong District, Tianjin, 300170 China; 2 Department of Gastroenterology and Hepatology, Tianjin Third Central Hospital, Tianjin, 300170 China

**Keywords:** Posterior reversible encephalopathy syndrome, Chemoembolization, Hepatocellular carcinoma, Chemotherapy

## Abstract

**Background:**

Posterior reversible encephalopathy syndrome (PRES) is a very rare complication secondary to transcatheter arterial chemoembolization (TACE). Only two patients with liver metastasis have been reported. We report for the first time two cases of hepatocellular carcinoma (HCC) patients occurred PRES secondary toTACE.

**Case presentation:**

The two patients with HCC developed headache, epilepsy, expressive aphasia, visual impairment and loss of consciousness, 11 and 3 h after conventional TACE (c-TACE) surgery. One patient experienced raised blood pressure during and after TACE, accompanied by a significant elevated creatinine. The magnetic resonance imaging (MRI) of the two patients showed multiple abnormal signals in the brain, mainly located in the white matter region. Combined with the clinical symptoms and MRI findings, PRES was diagnosed. Their symptoms and MRI changes improved significantly in the next two weeks.

**Conclusion:**

The PRES in this report is chemoembolization-associated syndrome, which might be related to the use of chemotherapy agents during TACE. And if neurological symptoms occur after TACE, patients should be closely monitored to exclude PRES.

## Background

Transcatheter arterial chemoembolization (TACE) is one of the main treatment methods for intermediate-stage liver cancer, and the most common post-operative complications are pain, nausea and fever [1]. Posterior reversible encephalopathy syndrome (PRES) was first reported by Hinchey et al. [2] in 1966 and is usually accompanied by clinical and neurological manifestations that can be visualized on radiological imaging [3]. It is a rare complication secondary to TACE, and only Pawar et al. [4] and Kistler et al. [5] have reported PRES after drug eluting beads (DEBs) TACE in patients with metastatic breast cancer to liver and metastatic uveal melanoma to liver in the past more than 50 years, respectively. In this paper, we reported two cases of hepatocellular carcinoma (HCC) patients with PRES accompanied by conventional TACE (c-TACE) for the first time and attempted to analyze its pathogenesis.

## Case presentation

**Fig. 1:**
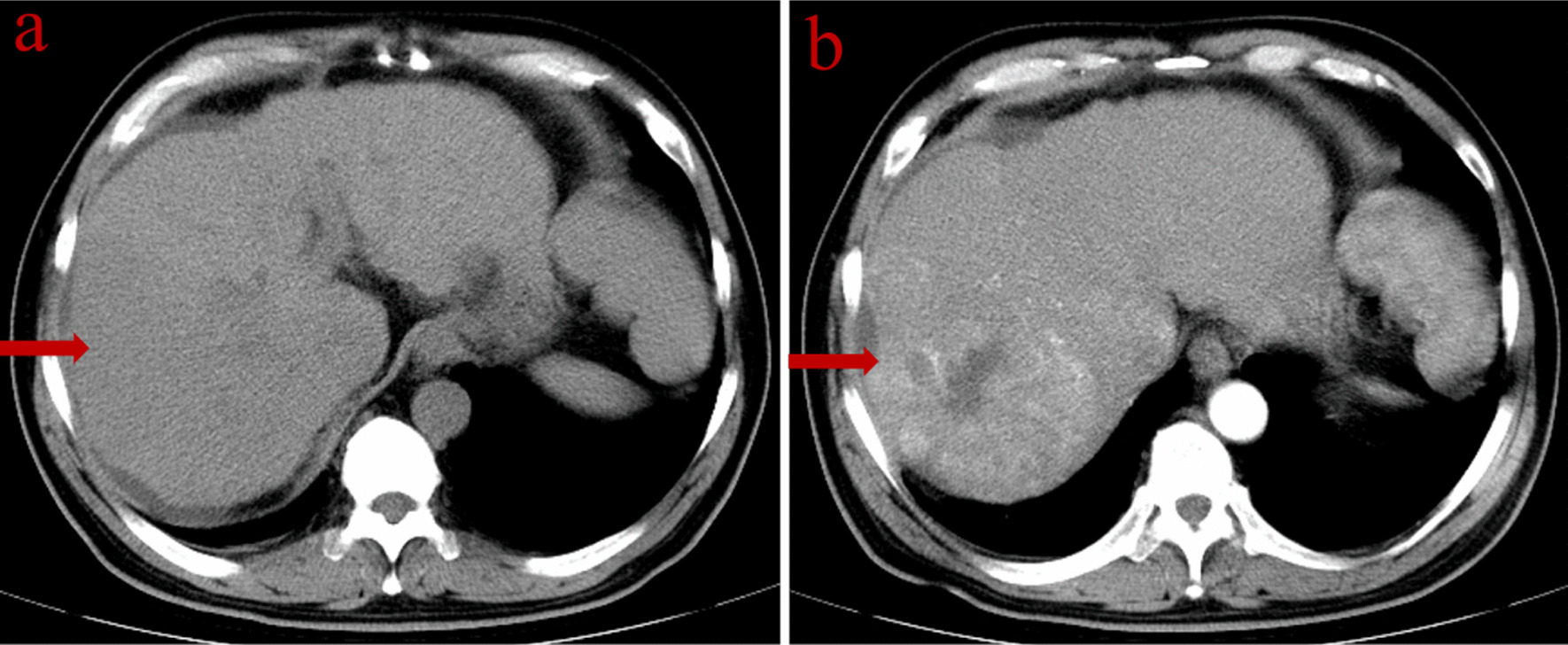
**a** CT scan showed an irregular low-density soft tissue mass in S6. **b** In the arterial phase, the mass was enhanced homogeneously, with a size of approximately 6.5 cm × 5.8 cm

**Fig. 2:**
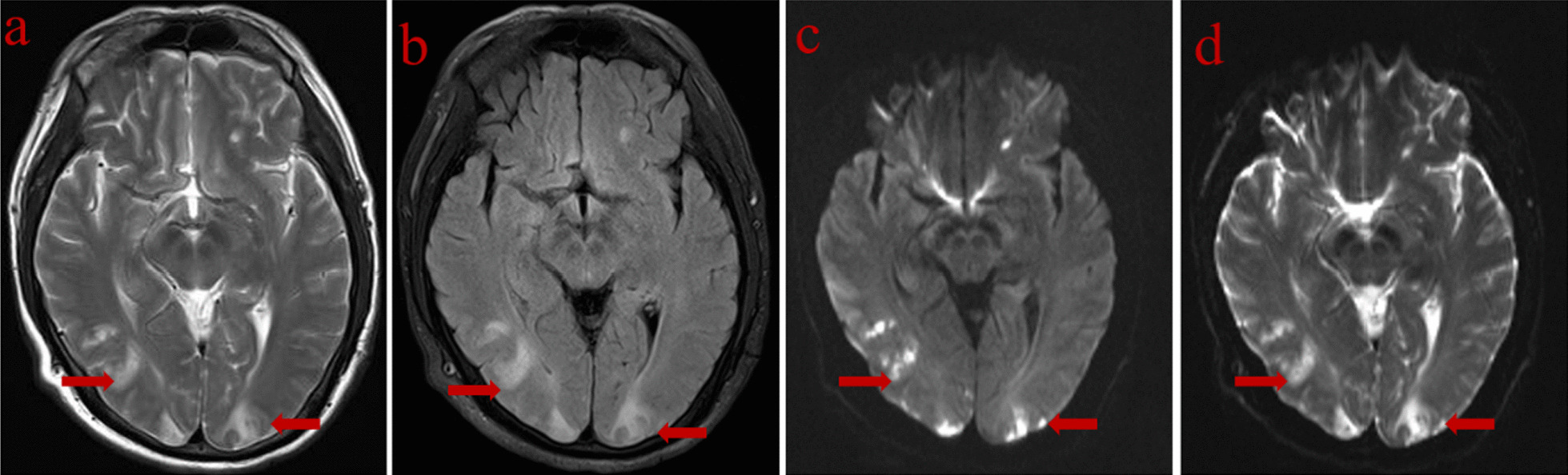
The brain MRI scan showed multiple patchy abnormal signal in the bilateral temporal lobe and the bilateral occipital lobe. T1WI (**a**),T2-FLAIR (**b**), DWI (**c**), ADC (**d**)

**Fig. 3:**
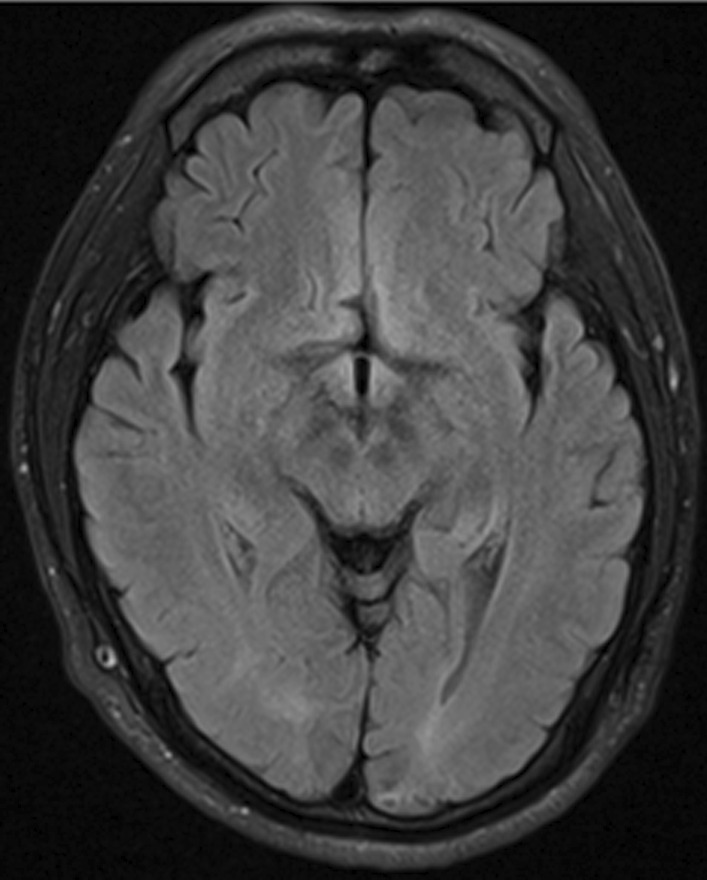
The brain MRI scan after 15 days. No obvious abnormal signal on T2-FLAIR

From January 2000 to March 2017, a total of 23,000 patients with hepatocellular carcinoma diagnosed clinically or pathologically received TACE treatment in our hospital, and two patients of these had accompanying PRES after c-TACE. Case 1 was a 47-year-old male patient with a history of alcoholic cirrhosis. Height 180 cm, weight 77 kg, BMI 23.8. His vital signs and laboratory tests were within the normal range, liver function classification was Child A, and the model for end-stage liver disease (MELD) score was seven. Echocardiography showed no right-left shunt (e.g., patent foramen ovale). No preoperative chemotherapy was given. Lumpy tumor staining in the right lobe of the liver, and no artery-venous fistula was observed in the first intraoperative angiography of TACE. 1000 mg of 5-fluorouracil and 300 mg of calcium folinate were perfused through the proper hepatic artery for chemotherapy. Then the mixed emulsion of 7 ml iodized oil and 20 mg epirubicin, and gelatin sponge particles were injected into the tumor arteries superselective through microcatheter for embolization. The formulation of chemotherapy scheme is based on EASL hepatocellular carcinoma management guidelines [6] and Chinese guidelines for TACE of hepatocellular carcinoma [7], in which the mixed emulsion of iodized oil and epirubicin is prepared in the form of water in oil (WiO) [6, 8]. According to the calculation of conversion factors for effective dose for TACE procedures [9], the fluorescence time during TACE was 547 s, the cumulative air kerma was 407.58 mGy, and the cumulative dose area product (DAP) was 145 Gycm^2^. During the TACE, this patient presented nausea and vomiting. Epilepsy and expressive aphasia occurred 11 h after operation. The computed tomography (CT) of brain was normal, while magnetic resonance imaging (MRI) of brain showed multiple abnormal signal in the white matter areas of the parietal, occipital and frontal lobes. On T2 weighted images (T2WI), T2 fluid attenuated inversion recovery (FLAIR), diffusion-weighted images (DWI) and apparent diffusion coefficient (ADC) maps showed hyper-intensity. (showed in Fig. 1). Laboratory tests showed that aspartate transaminase (AST) (108 U/L) and alanine aminotransferase (ALT) (59 U/L) were increased (the pre-treatment values of AST and ALT were 26 U/L and 30 U/L, respectively), and total bilirubin (TBIL) (8 μmol/L) was normal (Figs. 2, 3).

Case 2 was a 53-year-old female patient with a history of hepatitis B and cirrhosis, chronic renal insufficiency for 8 years, and hypertension for 20 years with a maximum of 178/72 mmHg. Height 158 cm, weight 44 kg, BMI 17.6. She did not take antihypertensive drugs regularly for 2 months before this surgery, and her blood pressure fluctuated between 130/70 and 140/80 mmHg. Her liver function classification was Child B, the MELD score was nine, and creatinine level was 127 μmol/L. Echocardiography showed no right-left shunt (e.g., patent foramen ovale). The TACE treatment had been performed three times before, and the postoperative symptoms were abdominal pain, abdominal distension and nausea. In this TACE operation, 100 mg of oxaliplatin was injected into the proper hepatic artery for chemotherapy, and then microcatheter superselective was used to inject mixed emulsion of 5 ml iodized oil and 10 mg epirubicin, and gelatin sponge particles were injected into the tumor blood supply artery for embolization. The fluorescence time during TACE was 897 s, the cumulative air kerma was 675.83 mGy, and the cumulative DAP was 230 Gycm^2^. Her blood pressure elevated to 152/78 mmHg during this time, headache and loss of consciousness occurred 3 h after the operation. And the binocular vision was seriously reduced. Physical examination showed that the pupillary light reflex, eye movements and fundus oculi were normal. The CT of brain was normal. MRI of brain found multiple abnormal signal in the parietal lobe, occipital lobe and temporal lobe, showed hyperintensity on T2WI, FLAIR, DWI, and ADC maps. The liver function test showed that AST (121 U/L) and ALT (89 U/L) increased (the pre-treatment values of AST and ALT were 71 U/L and 21 U/L, respectively), and TBIL was 17 μmol/L, and creatinine was 269 μmol/L. Blood pressure elevated to172/99 mmHg 5 h after TACE.

According to the clinical history, clinical manifestation, and imaging findings, combined with the diagnostic criteria proposed by Fugate and Rabinstein [7], PRES was the first diagnosis for the two patients. In case one, the clinical symptoms disappeared three days after liver protection, anti epilepsy and improvement of brain metabolism. The brain MRI showed that the abnormal signals basically disappeared two weeks later. After that, he received TACE treatments three times again within 10 months, but noPRES any more. In case 2, 13 h after controlling blood pressure, improving renal function and giving methylprednisolone to prevent inflammation and edema, the visual acuity recovered significantly. And the neurological symptoms disappeared completely 5 days later. After 12 days, creatinine decreased to 139 μmol/L. Brain MRI showed that the abnormal signals decreased significantly. No TACE treatment was received thereafter.

## Discussion and conclusion

The common complications after TACE are post embolism syndrome (PES), liver and kidney failure, liver abscess, cholesteatoma, ectopic embolism and so on [10]. The two patients reported in this paper had PES symptoms after operation, namely fever, nausea, vomiting, liver pain, abdominal distension and so on. The symptoms were relieved after symptomatic support therapy. Besides, transaminase increased in both patients, considering that transaminase is produced by hepatocytes and hepatocyte derived tumor cells, the application of superselective technology during TACE and the increase of transaminase after TACE do not meet the deterioration standard of liver function reserve, so the increase of serum concentration of these enzymes is mainly caused by tumor [11]. We think that the role of normal liver tissue cell necrosis in causing PRES is very small, and the follow-up CT did not show the signs of normal liver tissue infarction. PRES is an extremely rare complication after TACE. PRES refers to a group of clinical and imaging syndromes characterized by headache, nausea, visual disturbance, unconsciousness and epilepsy [12, 13]. The lesions are mainly located in the posterior cerebral white matter. On MRI, T1WI shows isointensity or slightly hypointensity, T2WI and FLAIR show hyperintensity, DWI shows isointensity or slightly hypointensity, and ADC maps shows hyperintensity suggesting that the lesions are angiogenic edema rather than cerebral ischemic venereal change [3, 13]. The clinical manifestation and imaging findings of the two cases were in line with the relevant literature.

Hypertension, preeclampsia, eclampsia, renal insufficiency, the use of immunosuppressants or cytotoxic drugs, systemic lupus erythematosus and so on, these factors play key role in the pathogenesis of PRES. Although its exact pathophysiological mechanism is not clear, several studies have reported that it may be related to the hyperperfusion of cerebral blood flow and impairment of endothelial cell function [13, 14].

In the cases reported by Pawar et al. [3] and Kistler et al. [4], patients with liver metastasis occurred PRES secondary to DEB-TACE treatment. And doxorubicin was used for embolization as in our report the two cases. The occurrence of PRES is related to endothelial cell injury caused by the application of cytotoxic drugs such as cyclophosphamide and doxorubicin, and systemic chemotherapy has been reported in the past [15]. Besides doxorubicin, many previous literatures [16, 17] have reported that chemotherapy with 5-fluorouracil or/and oxaliplatin could cause PRES. Although the cases we report were treated with 5-fluorouracil or oxaliplatin, and epirubicin locally to the arterial system, the two patients did not receive chemotherapy for eight weeks before operation, but the specific changes in clinical symptoms and imaging findings were potentially strongly correlated with the time of local application of chemotherapy agents. Given the temporal relationship between the treatment with oxaliplatin or 5-fluorouracil, and epirubicin, the exposure to either (or both) of these drugs seems the most likely trigger for the development of PRES in the two cases. Even at reasonable doses, drugs can still reach and destroy the blood–brain barrier of the central nervous system, similar to systemic chemotherapy [4]. We speculate that the occurrence of PRES has a low correlation with the dose of chemotherapeutic drugs, however, there is no reliable evidence related to this in literatures. Previous studies have shown that significant increase in severe toxicity after chemoembolization was found [18], which is more supportive of PRES caused by chemotherapeutic drugs. The use of bland transarterial embolization (TAE) may avoid or at least reduce the occurrence of PRES, but there is no strong evidence to prove that TAE can reduce the occurrence of neurological complications, so we can only speculate that TAE can avoid or at least reduce the risk of these complications. Moreover, both Barcelona clinical liver cancer (BCLC) staging system [19] and EASL liver cancer management guidelines [6] recommend TACE rather than TAE as the first-line treatment for medium-term HCC, and doxorubicin is the most commonly used chemotherapy drug for TACE [18]. In addition, the patient of case 2 received TACE treatments 4 times, which may have a cumulative effect on the permeability of cerebral endothelial cells, and her chronic renal insufficiency may also result in PRES due to water and sodium retention, toxin excretion obstacles and other factors [20].

It has been reported that approximately 75% of patients with PRES have accompanying elevated blood pressure [12, 21], and some patients also develop hypertension after TACE [22, 23]. In the case reported by Kistler et al. [5], the highest blood pressure after DEB-TACE increased to 180/113 mmHg. In this report, the patient of case 2 developed hypertension during and after c-TACE. Another mechanism for the occurrence of PRES is the hyperperfusion theory, which is closely related to hypertension. Because of the sudden rise in systemic blood pressure and the dysfunction of cerebrovascular self-regulation, the expansion of cerebral blood vessels and the destruction of the blood–brain barrier lead to the extravasation of blood to the brain parenchyma and the formation of angiogenic brain edema. However, the posterior area of the brain is more susceptible to hyperperfusion caused by a sudden increase in blood pressure due to its scarce sympathetic fibers and is more likely to be affected. Therefore, the neurological symptoms and abnormal signals in brain are often significantly relieved after controlling for blood pressure [12]. It has also been reported that symptoms usually disappear within 2 weeks after blood pressure control [5], but without timely and correct treatment, some patients with PRES can become disabled or even die [24]. Thus, it is reasonable to believe that the use of doxorubicin during TACE and the elevated blood pressure after TACE may play a synergistic role in increasing the susceptibility of PRES.

In addition, the use of contrast agents during TACE may also play a role in PRES. The patient of case 2 also had the symptoms of visual impairment. According to the course and clinical manifestations of the disease, the contrast agent-related transient cortical blindness (TCB) was considered. TCB is a recognized complication after cerebral angiography [25], which is closely related to the dose and type of contrast agent. Newman et al. [26] reported that the incidence of TCB was 0.3–1.0% and can occur after angiography of the peripheral vascular, abdominal vascular and the spinal canal. The main clinical manifestations are partial or complete loss of vision, normal fundi, normal papillary reflex, and unchanged extraocular movement. Some scholars believe that TCB is a clinical manifestation of PRES, while the mechanism of its occurrence is not clear at present. It may have the same pathophysiological mechanism as PRES [27]. It is generally recognized that the damage to the blood–brain barrier and the direct neurotoxic effect of contrast agents [28, 29], that is, the injection of contrast agents into arteries, can open the tight connection between capillary endothelial cells and increase pinocytosis to cross the blood brain barrier and play a neurotoxic role. However, TCB was not associated with loss of consciousness in previous studies, and in combination with brain MRI findings of this patient, PRES was the first diagnosis.

As far as we know, the incidence of PRES after c-TACE for hepatocellular carcinoma is very low. Although the clinical manifestation and imaging findingsare relatively serious, patients can obtain a better prognosis after timely and correct treatment. With the widely application of TACE with doxorubicin (including c-TACE and DEBs-TACE) in the treatment of liver malignant tumors, while preventing and managing common complications is routine, clinicians need to be reminded to pay attention to the possibility of rare complications, such as PRES. In particular, when the patient has neurological symptoms, brain MRI should be performed in a timely manner to determine whether it is accompanied by PRES.

## Data Availability

The datasets used and/or analyzed during the current study are available from the corresponding author on reasonable request.

## Abbreviations

PRES: Posterior reversible encephalopathy syndrome
TACE: Transcatheter arterial chemoembolization
c-TACE: Conventional TACE
HCC: Hepatocellular carcinoma
DEBs: Drug eluting beads
CT: Computed tomography
MRI: Magnetic resonance imaging
MELD: Model for end-stage liver disease
T2WI: T2 weighted images
FLAIR: Fluid attenuated inversion recovery
DWI: Diffusion-weighted images
ADC: Apparent diffusion coefficient
AST: Aspartate transaminase
ALT: Alanine aminotransferase
TBIL: Total bilirubin
TCB: Transient cortical blindness

## References

[1] Xiang H, Long L, Yao Y (2019). CalliSpheres drug-eluting bead transcatheter arterial chemoembolization presents with better efficacy and equal safety compared to conventional TACE in treating patients with hepatocellular carcinoma. Technol Cancer Res Treat.

[2] Hinchey J, Chaves C, Appignani B (1996). A reversible posterior leukoencephalopathy syndrome. N Engl J Med.

[3] Liman TG, Bohner G, Heuschmann PU (2012). The clinical and radiological spectrum of posterior reversible encephalopathy syndrome: the retrospective Berlin PRES study. J Neurol.

[4] Pawar PS, Noviawaty I, Zaidat OO (2012). Unusual case of intra-arterial doxorubicin chemoembolization-associated posterior reversible encephalopathy syndrome. Neurologist.

[5] Kistler CA, Mccall JC, Ghumman SS (2014). Posterior reversible leukoencephalopathy syndrome secondary to hepatic transarterial chemoembolization with doxorubicin drug eluting beads. J Gastrointest Oncol.

[6] Galle PR, Forner A, Llovet JM (2018). EASL clinical practice guidelines: management of hepatocellular carcinoma. J Hepatol.

[7] Clinical Guidelines Committee of Chinese Interventionalists College. [Chinese clinical practice guidelines for transarterial chemoembolization of hepatocellular carcinoma]. Zhonghua Nei Ke Za Zhi. 2021, 60(7):599–614. 10.3760/cma.j.cn112137-20210425-00991 34619836

[8] Renzulli M, Peta G, Vasuri F (2021). Standardization of conventional chemoembolization for hepatocellular carcinoma. Ann Hepatol.

[9] Compagnone G, Giampalma E, Domenichelli S (2012). Calculation of conversion factors for effective dose for various interventional radiology procedures. Med Phys.

[10] Shi M, Li-Gong L, Wan-Qiang F (2013). Roles played by lipiodolization and embolization in chemoembolization for hepatocellular carcinoma: single-blind, randomized trial. J Natl Cancer Inst.

[11] Granito A, Facciorusso A, Sacco R (2021). TRANS-TACE: prognostic role of the transient hypertransaminasemia after conventional chemoembolization for hepatocellular carcinoma. J Pers Med.

[12] Fugate JE, Rabinstein AA (2015). Posterior reversible encephalopathy syndrome: clinical and radiological manifestations, pathophysiology, and outstanding questions. Lancet Neurol.

[13] Kalaiselvan MS, Renuka MK, Arunkumar AS (2017). Clinical features and outcomes of patients with posterior reversible encephalopathy syndrome. Indian J Crit Care Med.

[14] Toledano M, Fugate JE (2017). Posterior reversible encephalopathy in the intensive care unit. Handb Clin Neurol.

[15] Edwards MJJ, Walker R, Vinnicombe S (2001). Reversible posterior leukoencephalopathy syndrome following CHOP chemotherapy for diffuse large B-cell lymphoma. Ann Oncol.

[16] Truman N, Nethercott D (2013). Posterior reversible encephalopathy syndrome (PRES) after treatment with oxaliplatin and 5-fluorouracil. Clin Colorectal Cancer.

[17] Gandini J, Manto M, Charette N (2020). Delayed posterior reversible leukoencephalopathy syndrome triggered by FLOT chemotherapy. Front Neurol.

[18] Facciorusso A, Bellanti F, Villani R (2017). Transarterial chemoembolization vs bland embolization in hepatocellular carcinoma: a meta-analysis of randomized trials. United Eur Gastroenterol J.

[19] Forner A, Reig M, Bruix J (2018). Hepatocellular carcinoma. Lancet.

[20] Ishikura K, Ikeda M, Hamasaki Y (2008). Nephrotic state as a risk factor for developing posterior reversible encephalopathy syndrome in paediatric patients with nephrotic syndrome. Nephrol Dialys Transplant.

[21] Bartynski WS (2008). Posterior reversible encephalopathy syndrome, part 1: fundamental imaging and clinical features. Ajnr Am J Neuroradiol.

[22] Basile A, Carrafiello G, Ierardi AM (2012). Quality-improvement guidelines for hepatic transarterial chemoembolization. Cardiovasc Intervent Radiol.

[23] Tasneem AA, Abbas Z, Luck NH (2013). Adverse events following transarterial chemoembolization for hepatocellular carcinoma and factors predicting such events. J Pak Med Assoc.

[24] Hinduja A, Habetz K, Raina S (2016). Predictors of poor outcome in patients with posterior reversible encephalopathy syndrome. Int J Neurosci.

[25] Jackson A, Stewart G, Wood A (1995). Transient global amnesia and cortical blindness after vertebral angiography: further evidence for the role of arterial spasm. Am J Neuroradiol.

[26] Newman CB, Schusse C, Hu YC (2011). Acute transient cortical blindness due to seizure following cerebral angiography. World Neurosurg.

[27] Suzuki S, Tanigawa N, Kariya S (2011). Posterior reversible encephalopathy syndrome occurring after uterine artery embolization for uterine myoma. Cardiovasc Intervent Radiol.

[28] Baguma M, Younan N, London A, Frédéric S (2017). Contrast-associated transient cortical blindness: three cases with MRI and electrophysiology findings. Acta Neurol Bel.

[29] Sesar A, Cavar I, Sesar AP (2018). Transient cortical blindness in posterior reversible encephalopathy syndrome after postpartum eclampsia. Taiwan J Ophthalmol.

